# Reasons for discontinuing clozapine: A cohort study of patients commencing treatment

**DOI:** 10.1016/j.schres.2016.05.002

**Published:** 2016-07

**Authors:** Sophie E. Legge, Marian Hamshere, Richard D. Hayes, Johnny Downs, Michael C. O'Donovan, Michael J. Owen, James T.R. Walters, James H. MacCabe

**Affiliations:** a Division of Psychological Medicine and Clinical Neurosciences, MRC Centre for Neuropsychiatric Genetics and Genomics, School of Medicine, Cardiff University, Cardiff, UK; b Department of Psychological Medicine, Institute of Psychiatry, Psychology and Neuroscience, King's College London, UK; c Department of Psychosis Studies, Institute of Psychiatry, Psychology and Neuroscience, King's College London, UK

**Keywords:** Clozapine, Schizophrenia, Treatment-resistant schizophrenia, Discontinuation, Cessation

## Abstract

**Background:**

Clozapine is uniquely effective in the management of treatment-resistant schizophrenia (TRS). However, a substantial proportion of patients discontinue treatment and this carries a poor prognosis.

**Methods:**

We investigated the risk factors, reasons and timing of clozapine discontinuation in a two-year retrospective cohort study of 316 patients with TRS receiving their first course of clozapine. Reasons for discontinuation of clozapine and duration of treatment were obtained from case notes and Cox regression was employed to test the association of baseline clinical factors with clozapine discontinuation.

**Results:**

A total of 142 (45%) patients discontinued clozapine within two years. By studying the reasons for discontinuations due to a patient decision, we found that adverse drug reactions (ADRs) accounted for over half of clozapine discontinuations. Sedation was the most common ADR cited as a reason for discontinuation and the risk of discontinuation due to ADRs was highest in the first few months of clozapine treatment. High levels of deprivation in the neighbourhood where the patient lived were associated with increased risk of clozapine discontinuation (HR = 2.12, 95% CI 1.30–3.47).

**Conclusions:**

Living in a deprived neighbourhood was strongly associated with clozapine discontinuation. Clinical management to reduce the burden of ADRs in the first few months of treatment may have a significant impact and help more patients experience the benefits of clozapine treatment.

## 1 Background

The superior efficacy of clozapine has been consistently demonstrated for those with treatment-resistant schizophrenia (TRS) (Kane et al., 1988, Leucht et al., 2009). Clozapine therapy has also been associated with decreased rates of mortality (Hayes et al., 2015), suicide (Meltzer et al., 2003, Tiihonen et al., 2009) and aggression (Chengappa et al., 2002). However, approximately 40% of patients will discontinue clozapine treatment within 24 months of initiation (Ciapparelli et al., 2000, Davis et al., 2014, Whiskey et al., 2003), and this is often followed by a rapid deterioration (Seppala et al., 2005), increased rates of compulsory treatment, re-hospitalisation, and poorer functioning (Atkinson et al., 2007, Wheeler et al., 2009).

Given the benefits of clozapine treatment and the poor prognosis for those who discontinue, efforts have been made to identify patients that may be at increased risk of discontinuation and to understand the causes. An older age at clozapine initiation, Black African/Caribbean ethnicity and substance abuse have been found to be associated with clozapine discontinuation (Davis et al., 2014, Krivoy et al., 2011, Moeller et al., 1995). The most common reasons for discontinuation identified in previous studies were patient decision, non-adherence and adverse drug reactions (Atkinson et al., 2007, Davis et al., 2014, Mustafa et al., 2015, Pai and Vella, 2012, Taylor et al., 2009). Although patient decision and non-adherence have been identified as major reasons for discontinuation of clozapine, there has been no exploration of reasons behind this choice.

The majority of previous studies have not been conducted in patients receiving their first trial of clozapine and thus the identified reasons for discontinuing may have been biased by previous clozapine trials. In the current study, we investigated the risk factors, reasons and timing of clozapine discontinuation in a two-year retrospective cohort study of all patients starting their first clozapine trial over a five-year period (2007–2011, inclusive) in South London and Maudsley (SLaM) NHS Foundation Trust.

## 2 Method

### 2.1 Setting

The study used data from the Clinical Records Interactive Search (CRIS) system; a large, anonymised case register derived from South London and Maudsley (SLaM) NHS Foundation Trust electronic case records and fully described elsewhere (Fernandes et al., 2013, Stewart et al., 2009). The CRIS system allows researchers to search structured and free text fields. SLaM is the largest secondary mental health care provider in Europe serving approximately 1.2 million people from four London boroughs; Lambeth, Croydon, Lewisham and Southwark.

### 2.2 Sample inclusion criteria

The cohort consisted of patients who had a lifetime ever ICD-10 primary diagnosis of a psychotic disorder (F20–F29, inclusive) and who began their first trial of clozapine between 1 January 2007 and 31 December 2011. This study period was selected because electronic records were fully implemented during 2006 in SLaM and clozapine initiations on or before 31 December 2011 permitted a two-year follow-up to the time of data extraction (January 2014). Patients were aged 18–65 years at the start of clozapine treatment and initiated clozapine under standard secondary mental health care services, either as an inpatient or outpatient. Patients who received tertiary care from SLAM national services were excluded because complete follow-up data were not always available and they were not a representative sample.

**Fig. 1:**
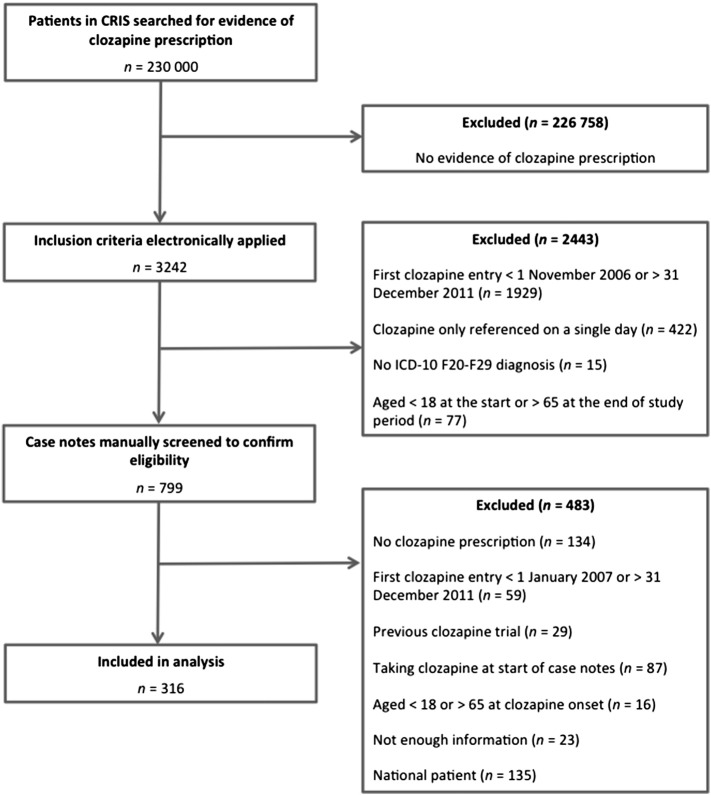
**Process of sample identification.** The sample was initially extracted using a general architecture for text engineering (GATE) application, which detected a total of 3242 patients where a prescription of clozapine was indicated. Patients were then selected whose first clozapine entry was between 1 November 2006 and 31 December 2011, had entries that spanned more than a single day, had a lifetime ever ICD-10 F20–F29 diagnosis, and aged 18 years or greater on 31 December 2011 and 65 years or less on 1 January 2007. The data for the 799 patients that met these criteria were manually screened and study eligibility verified from case notes.

The process of cohort identification is detailed in Fig. 1. A natural language processing application built using general architecture for text engineering (GATE) identified 3242 patients, from approximately 230,000 plus represented in CRIS, who had any evidence of current or previous clozapine use. The application used multiple data sources to identify medication use including pharmacy dispensing events, structured medication field, clinical correspondence and free text entries, resulting in a high degree of sensitivity (Hayes et al., 2015). We then selected patients who had (i) first clozapine prescription between 1 November 2006 (extended due to discussion that precedes clozapine initiation) and 31 December 2011, (ii) ICD-10 F20–F29 diagnosis, and (iii) aged 18 years or over on 31 December 2011 and 65 years or less on 1 January 2007. The data for the 799 patients who met these criteria were manually screened and study eligibility verified from their electronic clinical records.

### 2.3 Outcome measure

The timing and the reasons for clozapine discontinuation were assessed in a case note review for all patients who stopped treatment within 24 months of initiation. The date of clozapine initiation was defined as the date the patient took their first dose of clozapine. The date of discontinuation was defined as the date the patient was last known to take clozapine, where this was followed by at least three consecutive months without clozapine treatment.

Reasons for discontinuation were obtained from descriptive case notes when explicitly stated by the patient's clinical team. These were categorised into mutually exclusive reasons consistent with the previous literature (Atkinson et al., 2007, Davis et al., 2014, Mustafa et al., 2015, Pai and Vella, 2012, Taylor et al., 2009). If there were multiple reasons that spanned more than one category (of which there were only five instances), the most likely primary reason was inferred after discussion between first and corresponding author (Consultant Psychiatrist). Reasons for discontinuation were coded into categories of; (i) *adverse drug reaction* (ADR) defined as any unwanted or harmful reaction attributed to clozapine including intolerable side effects, (ii) *non-adherence not otherwise specified* defined as the patient declining to take medication, not attending for blood monitoring, or missing doses without informing their clinical team and with no reason for doing so stated (iii) *inadequate response* defined as insufficient improvement in symptoms, (iv) *blood monitoring* defined as a dislike of either blood tests or burden of frequent clinic visits, (v) *belief medication not required* defined as a patient belief that clozapine would not help them or that they did not need any medication, (vi) *delusional belief* held by the patient specifically regarding clozapine, (vii) *anticipated non-adherence* defined as pre-emptive discontinuation initiated by the clinical team as it was believed the patient would become non-adherent upon discharge from inpatient services, (viii) *death*, regardless of whether the cause was attributed to clozapine, and (ix) any *other* reason. If a patient discontinued due to non-adherence but cited a reason for doing so, they were classified under the reason given. To investigate differences in patients that were non-adherent, discontinuations were further classified as a *clinician-led decision* (defined as a discontinuation that was led by the clinical team, although in most cases this was a consensual decision between the patient and clinical team) or a *patient decision* (discontinuation due to non-adherence by declining to take medication, not attending for blood monitoring, or missing doses without informing their clinical team).

The specific ADR was recorded if it was stated to be the reason for discontinuation. These were not classified into mutually exclusive causes because in the majority of cases a number of ADRs were cited per patient and we wanted to reflect the broad adverse effect profile responsible for treatment discontinuation.

### 2.4 Exposure variables

Demographic details of age, gender, marital status, ethnicity, diagnosis, inpatient status and detention under the Mental Health Act were obtained from structured fields within CRIS. Age was defined as the patient's age at the date of clozapine initiation. Marital status was classified into *currently married/cohabiting* and *single*. Self-reported ethnicity was coded as *Black African/Caribbean* and *other*. The decision to aggregate into these categories was based on Black African/Caribbean ethnicity being the largest group within our sample, the relatively small cell counts of other ethnic groups, and trends reported in previous literature (Davis et al., 2014, Moeller et al., 1995). Diagnosis was classified into schizophrenia (ICD-10 code: F20) and non-schizophrenia F21–9 diagnosis (F21–F29, inclusive). The diagnosis closest in date to the start of clozapine was selected.

Level of deprivation was calculated from the 2007 Index of Multiple Deprivation (IMD) for England. The IMD is made up of seven individual measures of deprivation (income, employment, health deprivation and disability, education skills and training, barriers to housing and services, crime, and living environment) and is an established score for investigating social deprivation. The IMD score is calculated for geographical areas, which are ranked from one (most deprived) to 32,482 (least deprived). We arrived at cut-offs of deprivation ranks to give three roughly equal groups: high (1–5500), intermediate (5501–10,000) and low (10,001–32,482). The patient's home address closest to the start of the study period (1st January 2007) was used, with a separate category assigned to those who were homeless.

### 2.5 Analysis

We used a Kaplan-Meier survival curve to display the time to all-cause clozapine discontinuation and reasons for discontinuation. Having checked proportional hazard assumptions, a Cox regression was employed to model the association between all-cause clozapine discontinuation and gender, age, marital status, ethnicity, level of deprivation, diagnosis, inpatient status and detention under the Mental Health Act. Associations with all-cause discontinuation were assessed in a crude univariate analysis, and also in models that had been fully adjusted for all variables examined. Level of deprivation was entered into the model as a categorical dummy variable. We tested the appropriateness of entering age as a continuous variable with a likelihood ratio test. We investigated interaction effects with age, gender and ethnicity for variables significantly associated with all-cause discontinuation (P < 0.05). Sensitivity analyses were conducted whereby (i) death was classified as censored data rather than as a reason for all-cause discontinuation, and (ii) only patients with a diagnosis of schizophrenia (ICD-10 F20) were included. Competing-risks regression (Fine and Gray, 1999, Kim, 2007) was employed to model the impact of predictors on cause-specific discontinuation, whilst taking into account the other causes, firstly in a crude analysis and secondly, fully adjusted for all covariates examined. The specific causes of discontinuation investigated were; *ADRs*, *non-ADRs* (all reasons other than ADRs), *clinician-led decision* and *patient decision*. All statistical analyses were performed using STATA version 12 (StataCorp, 2011).

### 2.6 Ethical standards

Ethical approval for the use of CRIS as a research dataset was given by Oxfordshire Research Ethics Committee C (08/H0606/71) and the CRIS oversight committee granted permission for this study.

## 3 Results

### 3.1 Patient characteristics

A total of 316 patients were included in the study. Sample characteristics are presented in Table 1. The majority of the sample had a diagnosis of schizophrenia (n = 285) and the most common non-schizophrenia diagnosis was schizoaffective disorder (n = 21). In the *other* ethnicity category, 127 were White British, 17 Asian and 21 of other ethnicity. The majority of the 162 (51.3%) patients who were detained under the Mental Health Act at the time of clozapine initiation were under a section 3 (detention for treatment, n = 124) or sections 37–49 (forensic, n = 30).

**Table 1:** **Sample characteristics and risk for clozapine discontinuation.** Columns represent characteristics for total sample, those that discontinued and continued (reference group), hazard ratio and P-value from crude and fully adjusted Cox regression. Data for all 316 patients was available for each variable other than level of deprivation, which was available for 310 patients.

				Crude		Fully adjusted^a^	
Characteristic	Total sample (n = 316) n (%)	Discontinued (n = 142) n (%)	Continued (n = 174) n (%)	Hazard ratio (95% CI)	P-value	Hazard ratio (95% CI)	P-value
Male gender	205 (64.87)	89 (62.68)	116 (66.67)	0.86 (0.61–1.21)	0.381	0.77 (0.53–1.10)	0.155
Age at clozapine onset (years, SD)	36.23 (10.9)	36.11 (11.29)	36.33 (10.66)	1.00 (0.98–1.01)	0.717	0.99 (0.98–1.01)	0.423
Currently married or cohabiting	27 (8.54)	14 (9.86)	13 (7.47)	1.26 (0.73–2.19)	0.407	1.40 (0.76–2.57)	0.284
Black African/Caribbean ethnicity	151 (47.78)	81 (57.04)	70 (40.23)	1.55 (1.11–2.16)	0.010	1.26 (0.89–1.80)	0.194
Level of deprivation							
Low	84 (26.58)	26 (18.31)	58 (33.33)	Ref		Ref	
Intermediate	112 (35.44)	53 (37.32)	59 (33.91)	1.72 (1.08–2.75)	0.024	1.74 (1.06–2.83)	0.027
High	100 (31.65)	55 (38.73)	45 (25.86)	2.21 (1.38–3.52)	0.00091	2.12 (1.30–3.47)	0.0027
Homeless	14 (4.43)	6 (4.23)	8 (4.60)	1.44 (0.59–3.50)	0.419	1.51 (0.61–3.71)	0.373
Non-schizophrenia F21–9 diagnosis	31 (9.81)	14 (9.86)	17 (9.77)	1.01 (0.58–1.76)	0.966	0.82 (0.46–1.46)	0.497
Inpatient	262 (82.91)	115 (80.99)	147 (84.48)	0.84 (0.55–1.27)	0.401	0.66 (0.39–1.11)	0.119
Detained under Mental Health Act	162 (51.27)	81 (57.04)	81 (46.55)	1.32 (0.95–1.84)	0.104	1.34 (0.88–2.03)	0.168

Note: Follow-up period begins at start of clozapine treatment (from January 1, 2007 to December 31, 2011, inclusive) and ends with discontinuation, death or end of study period (24 months after treatment onset).

a Fully adjusted includes all variables.

### 3.2 Reasons for discontinuation

A total of 142 (45%) patients discontinued their first trial of clozapine within 24 months of initiation. Table 2 details the reasons for clozapine discontinuation. In total, 65 discontinuations (20.6% of all those starting clozapine) were from a *clinician-led decision* and 74 (23.4%) were from a *patient decision*. Three patients (0.9%) died within the study period. The majority of discontinuations from a *clinician-led decision* were due to *ADRs* (n = 54). We were able to obtain reasons for 49 of the 74 discontinuations due to a *patient decision* and thus the remaining 25 patients were classified as *non-adherence NOS*. *ADRs* were the most common reason for discontinuation from a *patient decision* (n = 26), followed by a *dislike of blood monitoring* (n = 10). Combined, *ADRs* attributed to clozapine were responsible for over half of the total discontinuations (n = 80). Discontinuations due to *blood monitoring* (n = 11) and an *inadequate response* (n = 8) were more frequent for *patient* than *clinician-led* discontinuations.

**Table 2:** **Reasons for clozapine discontinuation.** Columns represent discontinuations resulting from a *clinician-led decision*, *patient decision*, and combined total reasons. Percentages relate to all patients starting clozapine (n = 316).

Reason for discontinuation	Clinician-led decision n (%)	Patient decision n (%)	Combined n (%)
Adverse drug reaction	54 (17.1)	26 (8.2)	80 (25.3)
Non-adherence NOS	–	25 (7.9)	25 (7.9)
Blood monitoring	1 (0.3)	10 (3.2)	11 (3.5)
Inadequate response	3 (0.9)	5 (1.6)	8 (2.5)
Belief medication not required	0 (0.0)	4 (1.3)	4 (1.3)
Delusional belief	0 (0.0)	4 (1.3)	4 (1.3)
Anticipated non-adherence	2 (0.6)	0 (0.0)	2 (0.6)
Other	5 (1.6)	0 (0.0)	5 (1.6)
Death	–	–	3 (0.9)
Total	65 (20.6)	74 (23.4)	142 (44.9)

### 3.3 Time to clozapine discontinuation

**Fig. 2:**
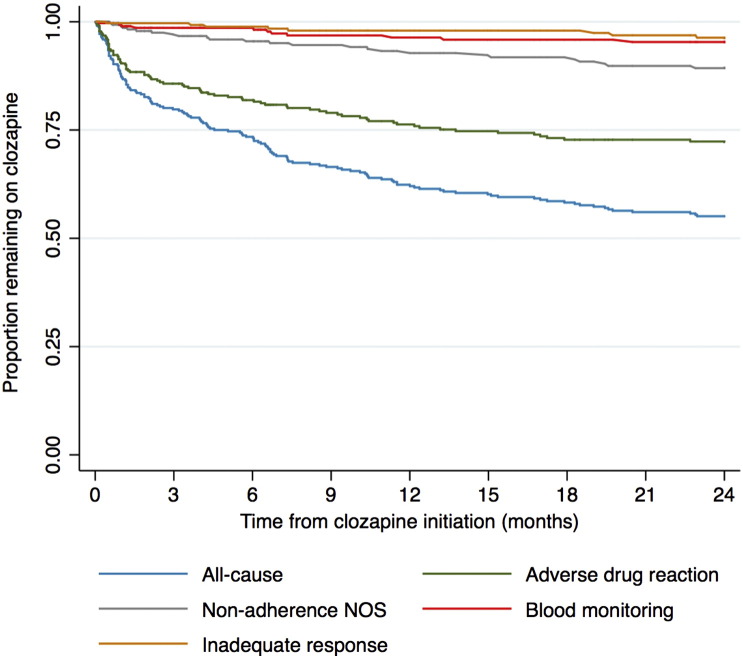
**Time to clozapine discontinuation.** Kaplan-Meier survival curve demonstrating proportion remaining on clozapine over initial 24 months of clozapine treatment. Blue line represents all-cause discontinuation. Other lines represent discontinuations due to adverse drug reactions (green), non-adherence not otherwise specified (grey), blood monitoring (red) and inadequate response (orange).

Fig. 2 displays a Kaplan-Meier survival curve for the time to all-cause clozapine discontinuation and for overall reasons of *ADRs*, *non-adherence NOS*, *blood monitoring* and *inadequate response*. Due to the small number of observations, the timings of discontinuations due to reasons of a *belief medication is not required*, *delusional belief*, *anticipated non-adherence*, *death* and *other* were not displayed. Supplementary Table 1 gives the timings for all combined reasons. A substantial proportion of those who initiated clozapine discontinued within the first few months: 12.3% within one month, 20% within three months and 38% within a year. The mean time to all-cause discontinuation was 5.9 months and the median 4.0 months (analysis restricted to those that discontinued within 24 months). The risk of discontinuations due to *ADRs* was highest in the first three months of clozapine treatment (Fig. 2). By contrast, the risk of discontinuation due to *non-adherence NOS*, *blood monitoring* and *inadequate response* were evenly distributed across the study period.

In a comparison of all-cause clozapine discontinuation timings of *clinician-led* and *patient decisions* (Supplementary Fig. 1, Supplementary Table 2 and Supplementary Table 3), we observed that the risk in the first three months of treatment was higher for *clinician-led* than *patient* discontinuations.

### 3.4 Adverse drug reactions

**Fig. 3:**
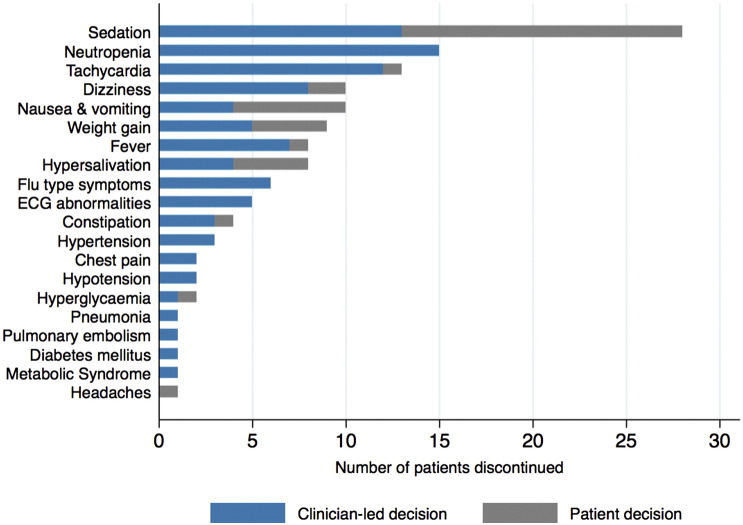
**Adverse drug reactions (ADRs)** cited as a reason for discontinuation of clozapine for 80 patients (130 ADRs). ADRs are not exclusive and differentiated by whether the discontinuation was a clinician-led decision (blue) or a patient decision (grey).

The 80 patients who discontinued clozapine due to *ADRs* cited a total of 130 individual ADRs. Fig. 3 displays the proportion of discontinuations due to ADRs that were from a *clinician-led* or *patient decision* (frequencies listed in Supplementary Table 4). Overall, sedation (n = 28), neutropenia (n = 15) and tachycardia (n = 13) were the most common ADRs cited as a reason for discontinuation of clozapine. The most common ADRs cited for *clinician-led* discontinuations were neutropenia (n = 15), sedation (n = 13), tachycardia (n = 12) and dizziness (n = 8). The most common ADR cited as a reason for discontinuation from a *patient decision* was sedation (n = 15), followed by nausea (n = 6), hypersalivation (n = 4) and weight gain (n = 4).

### 3.5 Risk factors for discontinuation

Table 1 details the association of predictors with all-cause clozapine discontinuation. In the fully adjusted model, intermediate (hazard ratio (HR) = 1.74, 95% CI 1.06–2.83) and high neighbourhood deprivation (HR = 2.12, 95% CI 1.30–3.47) were associated with increased risk for all-cause clozapine discontinuation. Black African/Caribbean ethnicity was associated with all-cause discontinuation in the crude analysis (HR = 1.55, 95% CI 1.11–2.16) but the association attenuated when fully adjusted (HR = 1.26, 95% CI 0.89–1.80). Gender, age, marital status, diagnosis, inpatient status and detention under the Mental Health Act were not associated with all-cause clozapine discontinuation. No interaction effects were identified. There were no differences in sensitivity analyses where death was not classed as a cause of discontinuation (Supplementary Table 5). However, in a sensitivity analysis restricted to those with a schizophrenia diagnosis, initiating clozapine as an inpatient was associated with a reduced risk of discontinuation (HR = 0.53, 95% CI 0.31–0.90, Supplementary Table 6).

Competing-risks regressions were used to investigate risk for cause-specific discontinuations. The association of predictors with discontinuations due to *ADRs* and *non-ADRs* are detailed in Supplementary Table 7. There was a significant association between level of deprivation and *non-ADR* discontinuations, but this was no longer significant in the fully adjusted model. The association of predictors with discontinuation due to a *clinician-led decision* or *patient decision* was also investigated (Supplementary Table 8). High deprivation was significantly associated with *patient decision* discontinuations, in both the crude and fully adjusted models (HR = 2.17, 95% CI 1.11–4.24).

## 4 Discussion

In a retrospective cohort study, we found that 45% of patients discontinued their first trial of clozapine within 24 months of initiation. Adverse drug reactions (ADRs) were responsible for over half of clozapine discontinuations and the risk of discontinuations due to ADRs was highest in the first few months of clozapine treatment. Neighbourhood deprivation was associated with an increased risk of clozapine discontinuation.

### 4.1 Reasons for discontinuation

This is the first study to examine in detail the reasons for discontinuations due to a *patient decision* and distinguish them from *clinician-led* or joint decisions to discontinue. By studying these reasons, we found that *ADRs* accounted for over half of clozapine discontinuations. Our results suggest that the role of ADRs has been underestimated as previous studies have used a restricted number of categories for discontinuation (i.e. *patient choice* and *non-adherence*), with no studies categorising the underlying reasons (Davis et al., 2014, Krivoy et al., 2011, Mustafa et al., 2015, Pai and Vella, 2012, Taylor et al., 2009). Our results are consistent with studies that have shown a quarter to two thirds of non-adherence to other antipsychotics was attributable to ADRs (Fenton et al., 1997, Hudson et al., 2004).

Sedation was the most frequently cited adverse effect, accounting for 20% of all discontinuations. Interestingly, over half of discontinuations due to sedation were from a *patient decision*. This is an important finding since sedation is usually transient and can almost always be minimised by reducing the dose and/or titration rate of clozapine, adjusting the timing of the dose or partial substitution with less sedating drugs such as aripiprazole (Nair and MacCabe, 2014). Around 10% of patients who start clozapine are discontinuing for this reason; it is likely many could remain on clozapine if this adverse effect was more actively managed and monitored by the clinical team.

It has been suggested that many discontinuations due to other ADRs could be avoided (Nielsen et al., 2013), although the appropriateness of any given reason was not assessed in this study. Nonetheless, our findings suggest that prompt identification and appropriate management of ADRs has the potential to improve continuation of clozapine treatment.

Consistent with earlier reports (Davis et al., 2014, Mustafa et al., 2015, Pai and Vella, 2012, Taylor et al., 2009), discontinuation due primarily to an *inadequate response* to clozapine was rare, occurring in only 2.5% of patients. Given that non-response to clozapine has been estimated between 40 and 70% (Kane et al., 1988, Lieberman et al., 1994), this result is unlikely to reflect the true rates of non-response to clozapine but rather that non-response is seldom recorded as the primary reason to discontinue treatment. A patient (or clinician) may be more likely to tolerate an ADR and be willing to persevere with clozapine if they are experiencing a good clinical response to clozapine, but might instead discontinue clozapine, citing adverse effects, in the absence of a clinical response. Nevertheless, the small percentage of patients who discontinue primarily due to inadequate response is striking. It could be driven partly by concern over risk of further relapse upon cessation (Seppala et al., 2005) and partly by a lack of any other evidence-based treatment options.

An interesting and novel insight was the observation that discontinuation of clozapine due to a *dislike of blood monitoring* was reported in 3.5% of patients. This raises the question of whether rates would be higher in all those eligible for clozapine, an important issue given that low rates of clozapine prescription have been attributed to the burden of blood monitoring (Nielsen et al., 2010). In this cohort of patients initiating clozapine, three patients died (2% of discontinuations) during the follow-up period. This is in contrast with cross sectional studies of clozapine discontinuation, which reported death as accounting for 13% of clozapine discontinuations (Davis et al., 2014, Taylor et al., 2009).

### 4.2 Risk factors for discontinuation

As far as we are aware, this is the first study to observe an association between level of neighbourhood deprivation and risk of clozapine discontinuation. Furthermore, we found this result was driven by discontinuations resulting from a *patient decision*. Previous studies have reported mixed results regarding the relationship between socio-economic status and non-adherence to medication (Kane et al., 2013), although it is not measured in many studies. There is an established association between markers of social derivation (including at the neighbourhood level) and increased incidence of schizophrenia, but there is very limited further research exploring the nature and implications of this link (O'Donoghue et al., 2016). It is likely that social deprivation is a proxy marker for other factors that underlie discontinuation and non-adherence and therefore, the next stage should be to identify the causal factors that are acting in more deprived neighbourhoods that explain the association. These causal factors may include both individual level and wider health care related components. Individual level characteristics of these patient groups that may increase risk for clozapine discontinuation include comorbid substance abuse and chaotic social circumstances, which are associated with both neighbourhood deprivation and discontinuation (Krivoy et al., 2011). Alternatively, clinical teams supporting areas in high deprivation may be under increased pressure or have more limited resources. However, there is limited evidence to suggest that patients with schizophrenia living in more deprived neighbourhoods have different prescribing experiences to patients living in more affluent areas (Martin et al., 2014).

Consistent with previous studies, we observed increased rates of all-cause clozapine discontinuation in Black African/Caribbean patients (Davis et al., 2014, Moeller et al., 1995); 54% of Black African/Caribbean patients discontinued compared with 40% of non-Black African/Caribbean patients. However, this association attenuated and was not statistically significant after adjusting for other factors. We found no evidence to support previous findings that higher age at clozapine initiation increased risk for discontinuation (Davis et al., 2014, Krivoy et al., 2011, MacGillivray et al., 2003).

### 4.3 Study limitations

The primary limitation of this study is its retrospective nature, specifically that the quality of data available to determine reasons for discontinuation was limited to information entered into the electronic clinical records system by the patient's clinical team. However, benefits of this study design are that the results are reflective of routine clinical care and there was universal capture of patients commencing clozapine in a defined geographical area covering a population of 1.2 million people, with consequently little or no selection bias. The fact that informed consent was not required also eliminated the selection bias in favour of higher functioning patients that bedevils research on psychosis. Recall bias was minimised by the use of contemporaneous records and the minimal missing data allowed us to determine the reasons for discontinuation for all of the patients. Furthermore, CRIS incorporates routinely collected data from multiple sources, such as pharmacy dispensing information, to increase reliability. Although the use of IMD categories is a widely accepted approach to measure neighbourhood deprivation, it is possible that a postcode area may not be homogeneous and could contain varying levels of deprivation.

### 4.4 Conclusions

Considering that clozapine is the most effective treatment for TRS, it is important that avoidable discontinuation is minimised. By examining the reasons for discontinuations due to a patient decision we found that ADRs accounted for the majority of clozapine discontinuations. It is important that clinicians identify and treat ADRs attributed to clozapine, particularly in the first few months after treatment onset, before they lead to discontinuation. Patients who live in an area of high deprivation are at an increased risk of discontinuing clozapine and may need additional support to maintain engagement with treatment.

## Role of funding source

This work was supported by a Medical Research Council (MRC) PhD studentship to Sophie E Legge. Richard D Hayes is supported by an MRC Population Health Scientist Fellowship. Johnny Downs is supported by an MRC Clinical Research Training Fellowship. This project has received funding from the European Union Seventh Framework Programme for research, technological development and demonstration under grant agreement n° 279227. This publication reflects only the authors' views and the European Union is not liable for any use that may be made of the information contained therein.

CRIS is supported by the NIHR Biomedical Research Centre for Mental Health BRC Nucleus at the South London and Maudsley NHS Foundation Trust and Institute of Psychiatry, King's College London jointly funded by the Guy's and St Thomas' Trustees and the South London and Maudsley Trustees.

## Contributors

Sophie E Legge, James T R Walters and James H MacCabe designed the study. James T R Walters and James H MacCabe jointly supervised the project. Sophie E Legge undertook the statistical analysis and wrote the first draft of the manuscript. All authors advised on analysis, interpretation of the results and contributed to the manuscript.

## Conflict of interest

Richard D Hayes has received research funding from Roche, Pfizer, J&J and Lundbeck. Sophie E Legge, Marian Hamshere, Johnny Downs, Michael C O′Donovan, Michael J Owen, James TR Walters and James H MacCabe report no competing interests.

## Appendix A Supplementary data

Supplementary material. Image 1
