# Supplementary material for: Reasons for discontinuing clozapine: A cohort study of patients commencing treatment
Source: Schizophr Res. 2016 Jul;174(1-3):113–9. doi: 10.1016/j.schres.2016.05.002 (PMC5756540; doi:10.1016/j.schres.2016.05.002)
Supplement: Supplementary file 1 — Supplementary material. [file mmc1.docx]

# Supplementary Information

**Supplementary Figure 1:** Kaplan-Meier survival curve demonstrating cumulative timing of discontinuations due to a *clinician-led decision* (blue) or a *patient decision* (grey).

| **Month** | **N taking clozapine** | **ADR** | **Non-adherence NOS** | | **Inadequate response** | **Blood monitoring** | **Belief medication not required** | **Delusional belief** | **Anticipated non-adherence** | **Death** | **Other** |
| --- | --- | --- | --- | --- | --- | --- | --- | --- | --- | --- | --- |
| x ≤ 3 | 316 | 44 | 8 | 1 | | 4 | 2 | 2 | 0 | 1 | 2 |
| 3 > x ≤ 6 | 252 | 11 | 4 | 2 | | 0 | 1 | 0 | 0 | 0 | 2 |
| 6 > x ≤ 9 | 232 | 8 | 2 | 2 | | 4 | 1 | 0 | 2 | 2 | 1 |
| 9 > x ≤ 12 | 210 | 7 | 4 | 0 | | 1 | 0 | 2 | 0 | 0 | 0 |
| 12 > x ≤ 15 | 197 | 4 | 1 | 0 | | 1 | 0 | 0 | 0 | 0 | 0 |
| 15 > x ≤ 18 | 190 | 5 | 1 | 0 | | 0 | 0 | 0 | 0 | 0 | 0 |
| 18 > x ≤ 21 | 184 | 0 | 4 | 2 | | 1 | 0 | 0 | 0 | 0 | 0 |
| 21 > x ≤ 24 | 177 | 1 | 1 | 1 | | 0 | 0 | 0 | 0 | 0 | 0 |
| Total |  | 80 | 25 | 8 | | 11 | 4 | 4 | 2 | 3 | 5 |

**Supplementary Table 1:** Timing of combined reasons for discontinuation. Columns represent monthly interval from clozapine initiation, number of patients still taking clozapine at the beginning of each time interval and each reason for clozapine discontinuation.

| **Month** | **N taking clozapine** | **ADR** | **Inadequate response** | **Blood monitoring** | **Anticipated non-adherence** | **Other** |
| --- | --- | --- | --- | --- | --- | --- |
| x ≤ 3 | 316 | 36 | 0 | 1 | 0 | 2 |
| 3 > x ≤ 6 | 252 | 6 | 0 | 0 | 0 | 2 |
| 6 > x ≤ 9 | 232 | 3 | 1 | 0 | 2 | 1 |
| 9 > x ≤ 12 | 210 | 5 | 0 | 0 | 0 | 0 |
| 12 > x ≤ 15 | 197 | 1 | 0 | 0 | 0 | 0 |
| 15 > x ≤ 18 | 190 | 3 | 0 | 0 | 0 | 0 |
| 18 > x ≤ 21 | 184 | 0 | 1 | 0 | 0 | 0 |
| 21 > x ≤ 24 | 177 | 0 | 1 | 0 | 0 | 0 |
| Total |  | 54 | 3 | 1 | 2 | 5 |

**Supplementary Table 2:** Timing of clinician-led discontinuations. Columns represent monthly interval from clozapine initiation, number of patients still taking clozapine at the beginning of each time interval, and each reason for *clinician-led* discontinuations.

| **Month** | **N taking clozapine** | **ADR** | **Inadequate response** | **Blood monitoring** | **Belief medication not required** | **Delusional belief** | **Non-adherence NOS** |
| --- | --- | --- | --- | --- | --- | --- | --- |
| x ≤ 3 | 316 | 8 | 1 | 3 | 2 | 2 | 8 |
| 3 > x ≤ 6 | 252 | 5 | 2 | 0 | 1 | 0 | 4 |
| 6 > x ≤ 9 | 232 | 5 | 1 | 4 | 1 | 0 | 2 |
| 9 > x ≤ 12 | 210 | 2 | 0 | 1 | 0 | 2 | 4 |
| 12 > x ≤ 15 | 197 | 3 | 0 | 1 | 0 | 0 | 1 |
| 15 > x ≤ 18 | 190 | 2 | 0 | 0 | 0 | 0 | 1 |
| 18 > x ≤ 21 | 184 | 0 | 1 | 1 | 0 | 0 | 4 |
| 21 > x ≤ 24 | 177 | 1 | 0 | 0 | 0 | 0 | 1 |
| Total |  | 26 | 5 | 10 | 4 | 4 | 25 |

**Supplementary Table 3:** Timing of discontinuations due to a *patient* decision. Columns represent monthly interval from clozapine initiation, number of patients still taking clozapine at the beginning of each time interval, and each reason for discontinuation.

| **ADR** | **Clinician-led**  **decision**  **N** | **Patient**  **decision**  **N** | **Combined**  **N** |
| --- | --- | --- | --- |
| Sedation | 13 | 15 | 28 |
| Neutropenia | 15 | 0 | 15 |
| Tachycardia | 12 | 1 | 13 |
| Dizziness | 8 | 2 | 10 |
| Nausea & vomiting | 4 | 6 | 10 |
| Weight gain | 5 | 4 | 9 |
| Fever | 7 | 1 | 8 |
| Hypersalivation | 4 | 4 | 8 |
| Flu type symptoms | 6 | 0 | 6 |
| ECG abnormalities | 5 | 0 | 5 |
| Constipation | 3 | 1 | 4 |
| Hypertension | 3 | 0 | 3 |
| Chest pain | 2 | 0 | 2 |
| Hypotension | 2 | 0 | 2 |
| Hyperglycaemia | 1 | 1 | 2 |
| Pneumonia | 1 | 0 | 1 |
| Pulmonary embolism | 1 | 0 | 1 |
| Diabetes mellitus | 1 | 0 | 1 |
| Metabolic Syndrome | 1 | 0 | 1 |
| Headaches | 0 | 1 | 1 |

**Supplementary Table 4:** Adverse drug reactions (ADRs) cited as a reason for discontinuation of clozapine (80 patients, a total of 130 ADRs). ADRs are not exclusive and differentiated by whether the discontinuation was from a *clinician-led decision* or *patient decision*.

|  | **Crude** | |  | **Fully Adjusted^1^** | |
| --- | --- | --- | --- | --- | --- |
| **Characteristic** | **Hazard ratio**  (95% CI) | **P-value** |  | **Hazard ratio**  (95% CI) | **P-value** |
| Male gender | 0.88 (0.63-1.25) | 0.487 |  | 0.79 (0.55-1.14) | 0.210 |
| Age at clozapine onset (years, SD) | 0.99 (0.98-1.01) | 0.620 |  | 0.99 (0.98-1.01) | 0.422 |
| Currently married or cohabiting | 1.19 (0.67-2.11) | 0.547 |  | 1.32 (0.70-2.47) | 0.394 |
| Black African/Caribbean ethnicity | 1.53 (1.10-2.15) | 0.013 |  | 1.27 (0.88-1.81) | 0.197 |
| *Level of deprivation* |  |  |  |  |  |
| Low | Ref |  |  | Ref |  |
| Intermediate | 1.72 (1.08-2.75) | 0.023 |  | 1.71 (1.05-2.79) | 0.032 |
| High | 2.09 (1.30-3.34) | 0.0023 |  | 1.98 (1.20-3.25) | 0.0071 |
| Homeless | 1.44 (0.59-3.51) | 0.418 |  | 1.49 (0.61-3.68) | 0.384 |
| Non-schizophrenia F21-9 diagnosis | 1.04 (0.60-1.80) | 0.899 |  | 0.85 (0.48-1.52) | 0.588 |
| Inpatient | 0.81 (0.54-1.24) | 0.338 |  | 0.64 (0.38-1.08) | 0.092 |
| Detained under Mental Health Act | 1.31 (0.93-1.83) | 0.117 |  | 1.36 (0.89-2.08) | 0.152 |

**Supplementary Table 5:** Sensitivity analysis for clozapine discontinuation including death as censored data rather than a reason for discontinuation. Columns represent Cox regression hazard ratio and P-value from crude and fully adjusted Cox regression. ^1^Fully adjusted includes all variables. Data for all 316 patients was available for each variable other than level of deprivation, which was available for 310 patients.

Note: Follow-up period begins at start of clozapine treatment (from January 1, 2007 to December 31, 2011, inclusive) and ends with discontinuation, death or end of study period (24 months after treatment onset).

|  | **Crude** | |  | **Fully Adjusted^1^** | |
| --- | --- | --- | --- | --- | --- |
| **Characteristic** | **Hazard ratio**  (95% CI) | **P-value** |  | **Hazard ratio**  (95% CI) | **P-value** |
| Male gender | 0.84 (0.58-1.20) | 0.341 |  | 0.73 (0.50-1.08) | 0.112 |
| Age at clozapine onset (years, SD) | 0.99 (0.98-1.01) | 0.467 |  | 0.99 (0.97-1.01) | 0.295 |
| Currently married or cohabiting | 1.22 (0.66-2.26) | 0.536 |  | 1.46 (0.73-2.91) | 0.279 |
| Black African/Caribbean ethnicity | 1.67 (1.17-2.38) | 0.0046 |  | 1.34 (0.92-1.95) | 0.128 |
| *Level of deprivation* |  |  |  |  |  |
| Low | Ref |  |  | Ref |  |
| Intermediate | 1.91 (1.16-3.14) | 0.011 |  | 1.99 (1.18-3.33) | 0.0093 |
| High | 2.44 (1.49-4.01) | 0.00043 |  | 2.32 (1.38-3.90) | 0.0014 |
| Homeless | 1.70 (0.69-4.19) | 0.245 |  | 1.78 (0.71-4.46) | 0.216 |
| Inpatient | 0.70 (0.45-1.07) | 0.103 |  | 0.53 (0.31-0.90) | 0.020 |
| Detained under Mental Health Act | 1.27 (0.90-1.80) | 0.180 |  | 1.37 (0.88-2.14) | 0.167 |

**Supplementary Table 6:** Sensitivity analysis for clozapine discontinuation only including patients with a schizophrenia diagnosis (ICD-10 F20) (n=285). Columns represent Cox regression hazard ratio and P-value from crude and fully adjusted Cox regression. ^1^Fully adjusted includes all variables. Data for all 285 patients was available for each variable other than level of deprivation, which was available for 279 patients.

Note: Follow-up period begins at start of clozapine treatment (from January 1, 2007 to December 31, 2011, inclusive) and ends with discontinuation, death or end of study period (24 months after treatment onset).

|  | **Crude** | |  | **Fully Adjusted^1^** | |
| --- | --- | --- | --- | --- | --- |
| **Characteristic** | **SHR**  (95% CI) | **P-value** |  | **SHR**  (95% CI) | **P-value** |
| ***i. Discontinuation due to ADRs*** |  |  |  |  |  |
| Male gender | 0.73 (0.47-1.14) | 0.169 |  | 0.66 (0.41-1.07) | 0.093 |
| Age at clozapine onset (years, SD) | 1.00 (0.98-1.02) | 0.829 |  | 0.99 (0.97-1.02) | 0.461 |
| Currently married or cohabiting | 1.74 (0.88-3.45) | 0.112 |  | 1.79 (0.79-4.04) | 0.164 |
| Black African/Caribbean ethnicity | 1.37 (0.88-2.13) | 0.161 |  | 1.13 (0.68-1.88) | 0.628 |
| *Level of deprivation* |  |  |  |  |  |
| Low | Ref |  |  | Ref |  |
| Intermediate | 1.70 (0.92-3.15) | 0.089 |  | 1.79 (0.90-3.55) | 0.097 |
| High | 1.66 (0.89-3.12) | 0.112 |  | 1.68 (0.84-3.34) | 0.141 |
| Homeless | 1.18 (0.35-3.91) | 0.790 |  | 1.36 (0.39-4.72) | 0.631 |
| Non-schizophrenia F21-9 diagnosis | 1.36 (0.71-2.63) | 0.353 |  | 1.06 (0.50-2.23) | 0.875 |
| Inpatient | 0.79 (0.46-1.37) | 0.411 |  | 0.63 (0.31-1.27) | 0.195 |
| Detained under Mental Health Act | 1.19 (0.76-1.84) | 0.445 |  | 1.27 (0.73-2.23) | 0.402 |
| ***ii. Discontinuations not due to ADRs*** | | | | | |
| Male gender | 1.15 (0.67-1.95) | 0.618 |  | 1.07 (0.60-1.91) | 0.815 |
| Age at clozapine onset (years, SD) | 1.00 (0.98-1.02) | 0.933 |  | 1.00 (0.98-1.03) | 0.905 |
| Currently married or cohabiting | 0.69 (0.23-1.83) | 0.457 |  | 0.88 (0.32-2.44) | 0.805 |
| Black African/Caribbean ethnicity | 1.65 (0.99-2.75) | 0.054 |  | 1.42 (0.84-2.41) | 0.191 |
| *Level of deprivation* |  |  |  |  |  |
| Low | Ref |  |  | Ref |  |
| Intermediate | 1.48 (0.72-3.04) | 0.286 |  | 1.37 (0.67-2.80) | 0.392 |
| High | 2.33 (1.16-4.69) | 0.017 |  | 2.06 (0.98-4.31) | 0.056 |
| Homeless | 1.71 (0.49-6.03) | 0.401 |  | 1.54 (0.44-5.41) | 0.504 |
| Non-schizophrenia F21-9 diagnosis | 0.61 (0.22-1.70) | 0.345 |  | 0.64 (0.21-1.93) | 0.424 |
| Inpatient | 0.96 (0.50-1.83) | 0.900 |  | 0.78 (0.35-1.76) | 0.553 |
| Detained under Mental Health Act | 1.43 (0.86-2.38) | 0.169 |  | 1.39 (0.72-2.69) | 0.327 |

**Supplementary Table 7:** Competing-risks regression to model impact of predictors on (i) discontinuation due to ADRs, whilst taking into account the other causes of discontinuation and (ii) discontinuation due to reasons other than ADRs, whilst taking into account discontinuations due to ADRs. SHR = subhazard ratio. ^1^Fully adjusted includes all variables. Data for all 316 patients was available for each variable other than level of deprivation, which was available for 310 patients.

|  | **Crude** | |  | **Fully Adjusted^1^** | |
| --- | --- | --- | --- | --- | --- |
| **Characteristic** | **SHR**  (95% CI) | **P-value** |  | **SHR**  (95% CI) | **P-value** |
| ***i. Clinician-led decision*** | | | | | |
| Male gender | 0.87 (0.53-1.43) | 0.589 |  | 0.87 (0.51-1.49) | 0.603 |
| Age at clozapine onset (years, SD) | 1.00 (0.98-1.03) | 0.765 |  | 1.00 (0.97-1.02) | 0.879 |
| Currently married or cohabiting | 2.18 (1.10-4.33) | 0.026 |  | 2.17 (0.95-4.95) | 0.067 |
| Black African/Caribbean ethnicity | 1.45 (0.89-2.36) | 0.131 |  | 1.26 (0.74-2.13) | 0.399 |
| *Level of deprivation* |  |  |  |  |  |
| Low | Ref |  |  | Ref |  |
| Intermediate | 1.67 (0.84-3.35) | 0.146 |  | 1.62 (0.76-3.48) | 0.213 |
| High | 1.73 (0.86-3.50) | 0.124 |  | 1.53 (0.71-3.31) | 0.277 |
| Homeless | 1.43 (0.43-4.74) | 0.561 |  | 1.68 (0.49-5.73) | 0.405 |
| Non-schizophrenia F21-9 diagnosis | 1.75 (0.89-3.42) | 0.102 |  | 1.43 (0.66-3.11) | 0.360 |
| Inpatient | 0.68 (0.38-1.21) | 0.188 |  | 0.48 (0.22-1.07) | 0.072 |
| Detained under Mental Health Act | 1.26 (0.78-2.05) | 0.341 |  | 1.56 (0.81-3.01) | 0.183 |
| ***ii. Patient decision*** | | | | | |
| Male gender | 0.91 (0.57-1.44) | 0.677 |  | 0.75 (0.46-1.25) | 0.270 |
| Age at clozapine onset (years, SD) | 0.99 (0.97-1.02) | 0.555 |  | 0.99 (0.97-1.02) | 0.604 |
| Currently married or cohabiting | 0.54 (0.21-1.41) | 0.210 |  | 0.66 (0.24-1.81) | 0.422 |
| Black African/Caribbean ethnicity | 1.51 (0.96-2.39) | 0.076 |  | 1.24 (0.75-2.07) | 0.403 |
| *Level of deprivation* |  |  |  |  |  |
| Low | Ref |  |  | Ref |  |
| Intermediate | 1.55 (0.82-2.91) | 0.176 |  | 1.59 (0.83-3.06) | 0.165 |
| High | 2.15 (1.15-4.00) | 0.016 |  | 2.17 (1.11-4.24) | 0.024 |
| Homeless | 1.40 (0.39-4.96) | 0.605 |  | 1.30 (0.34-4.89) | 0.701 |
| Non-schizophrenia F21-9 diagnosis | 0.47 (0.18-1.25) | 0.129 |  | 0.44 (0.15-1.25) | 0.122 |
| Inpatient | 1.11 (0.60-2.05) | 0.733 |  | 0.99 (0.48-2.02) | 0.975 |
| Detained under Mental Health Act | 1.31 (0.83-2.07) | 0.247 |  | 1.14 (0.64-2.01) | 0.661 |

**Supplementary Table 8:** Competing-risks regression to model impact of predictors on (i) discontinuations due to *clinician-led decision*, taking into account discontinuations due to *patient decision* and (ii) discontinuations due to *patient decision*, taking into account discontinuations from a *clinician-led decision*. SHR = subhazard ratio. ^1^Fully adjusted includes all variables. Data for all 316 patients was available for each variable other than level of deprivation, which was available for 310 patients.
